# New Insight into Nucleotide Changes on Irradiated *Bactrocera dorsalis* (Hendel), A Pest of Horticultural Importance

**DOI:** 10.21315/tlsr2024.35.2.14

**Published:** 2024-07-31

**Authors:** Suhana Yusof, Nurul Wahida Othman, Ahmad Zainuri Mohamad Dzomir, Muhamad Azmi Mohammed, Ameyra Aman-Zuki, Salmah Yaakop

**Affiliations:** 1 Horticulture Research Centre, Malaysian Agricultural Research and Development Institute (MARDI), MARDI Headquarters, Persiaran MARDI-UPM, 43400 Serdang, Selangor, Malaysia; 2 Centre for Insect Systematics, Department of Bioscience and Biotechnology, Faculty of Science and Technology, Universiti Kebangsaan Malaysia, 43600 Bangi Selangor, Malaysia; 3 Agrotechnology and Biosciences Division, Malaysian Nuclear Agency, 43000 Kajang, Selangor, Malaysia; 4 Department of Crop Science, Faculty of Agricultural and Forestry Sciences, Universiti Putra Malaysia Bintulu Sarawak Campus, Nyabau Road, 97008 Bintulu, Sarawak, Malaysia

**Keywords:** Commodity, *COI*, Gamma Radiation, Mitochondrial DNA, Pest, Preharvest, Postharvest, Quarantine, IPM, Komoditi, COI, Sinaran Gamma, DNA Mitokondria, Perosak, Pra tuaian, Lepas tuai, Kuarantin, IPM

## Abstract

*Bactrocera dorsalis* (Hendel) is a major quarantine pest species infesting most of the tropical fruits. Its infestation had significantly reduced and disrupted the export market trade, thus, very crucial to be controlled during the preharvest and postharvest. One of the most sustainable control methods is by using the radiation technique to reduce the pest population, thus curbing the spread of this pest to new geographical areas. The objective of this study was to measure the nucleotide changes in *B. dorsalis* (larval, pupal and adult stages) which had been irradiated with 50 to 400 Gray, using Gamma Cell Biobeam GM8000 irradiator with Cesium-137 source at the Malaysian Nuclear Agency, Selangor, Malaysia. Data from the treated samples (with and without morphological changes) were analysed using cytochrome oxidase subunit I (*COI*). The alignment of 59 sequences resulted in 0.92% variables with only four characters that were parsimony informative, and six sites (30, 60, 234, 282, 483 and 589) which had nucleotide changes, but had not been translated to another protein. Low polymorphism was presented on the sample groups, with only four haplotypes, but with high diversity value (Hd) = 0.5885. The phylogeny trees formed soft polytomy in both trees [neighbour joining (NJ) and maximum parsimony (MP)] presenting a mixture of individuals but did not show any significant difference between treatments. This finding concluded that low mutation had occurred on the treated *B. dorsalis* and this information is very valuable in getting new insight on the survival of *B. dorsalis* in the horticulture industry.

## Abstrak

*Bactrocera dorsalis* (Hendel) ialah spesies perosak kuarantin utama yang menyerang kebanyakan buah-buahan tropika. Serangannya telah mengurangkan dan mengganggu perdagangan pasaran eksport dengan ketara, justeru, pengawalan adalah sangat penting semasa pra tuai dan lepas tuai. Salah satu kaedah kawalan yang paling mampan ialah dengan menggunakan teknik sinaran untuk mengurangkan populasi perosak, sekali gus mengekang penularan perosak ini ke kawasan geografi yang baharu. Objektif kajian ini adalah untuk mengukur perubahan nukleotida dalam *B. dorsalis* (peringkat larva, pupal dan dewasa) yang telah disinari dengan 50 hingga 400 Gray, menggunakan penyinar Gamma Cell Biobeam GM8000 dengan sumber Cesium-137 di Agensi Nuklear Malaysia, Selangor, Malaysia. Data daripada sampel yang dirawat (dengan dan tanpa perubahan morfologi) telah dianalisis menggunakan subunit 1 cytochrome oxidase (*COI*). Penjajaran sebanyak 59 jujukan menghasilkan 0.92% pemboleh ubah dengan hanya empat ciri yang bermaklumat parsimoni, dan enam tapak (30, 60, 234, 282, 483, dan 589) yang mempunyai perubahan nukleotida, tetapi belum diterjemahkan kepada protein lain. Polimorfisme yang rendah telah dibentangkan pada kumpulan sampel, dengan hanya empat haplotip, tetapi dengan nilai kepelbagaian yang tinggi (Hd) = 0.5885. Pokok filogeni membentuk politomi lembut pada kedua-dua pokok (*Neighbour joining* (NJ) dan *Maximum parsimony* (MP)) yang membentangkan campuran individu tetapi tidak menunjukkan sebarang perbezaan yang ketara antara rawatan. Dapatan ini menyimpulkan bahawa mutasi rendah telah berlaku pada *B. dorsalis* yang dirawat dan maklumat ini sangat penting untuk mendapatkan pandangan baharu tentang kemandirian *B. dorsalis* dalam industri hortikultur.

**Highlights**

- Low polymorphism was presented on the treated sample groups, with only four haplotypes, but with high diversity value (Hd) = 0.5885.
- The phylogeny trees formed soft polytomy in both trees [neighbour joining(NJ) and maximum parsimony (MP)] presenting a mixture of individuals but did not show any significant difference between treatments.
- Low mutation had occurred on the treated *Bactrocera dorsalis* and this information is very valuable in getting new insight on the survival of *B. dorsalis* in the horticulture industry.

## INTRODUCTION

*Bactrocera dorsalis* (Hendel) is a major quarantine pest species infesting most of the tropical fruits in Asia, Australia and the South Pacific regions (Chua *et al*. 2009). The *B. dorsalis* infestation has significantly reduced and disrupted the export market trade (Stephens *et al*. 2007), which shows significant reduction in quality and productivity of the Malaysian fruit markets (Badri *et al*. 2008). The spread of *B. dorsalis* infestation is mainly due to its short life cycle, high reproductive rate and genetic changes, high mobility rate and high competition with the native species(Duyck *et al*. 2004; Vargas *et al*. 2007). Besides that, *B. dorsalis* has become one of the quarantine pests that are highly invasive and able to adapt to the new environment (CABI 2016).

*B. dorsalis* is a serious pest species in horticulture that must be controlled immediately throughout the preharvest and postharvest periods (Hallman 2012), and as a quarantine issue (Zainon & Lebai Juri 2000). One of the effective methods is by using the radiation technique, which is the most sustainable control method to reduce the pest population at preharvest stage with sterile insect technique (SIT), to control the spread of this pest to new geographical areas. The phytosanitary irradiation (PI) treatment is important in preventing the introduction of the quarantine pest species into the importing countries (Bustos-Griffin *et al*. 2015) by application of the optimum dose of gamma radiation that would not affect the irradiated quality of the fruit (Heather & Hallman 2008).

A few studies have been conducted on the effect of gamma radiation on the Malaysian *B. dorsalis*, for example, by Yusof *et al*. (2019), based on the biology (survivability and fecundity) and morphological differences in terms of ovary and testis sizes. However, the effect on their genetic attributes has never been investigated, eventhough it is crucial to understand the genetic changes for the management of pests to develop the new protocol for irradiation towards PI (Hallman 2004; Lance & McInnis 2005). The irradiation was also not affecting the chromosome number, as noted by Paithankar *et al*. (2017), but affected the gene expression, for example, on the YP gene in *Bactrocera tau* after post-emergence (Cai *et al*. 2018). Thus, it is crucial to determine the changes in *B. dorsalis* shown in their biology and physiology due to irradiation of gamma. It is because, the ecological and evolutionary consequences (Chown & Terblanche 2006).

Due to the various stages of insects acting as pests, accurate species identification is crucial in the application of quarantine, surveillance and biosecurity research. To overcome the problem, molecular approach is the best technique and has been proven to provide valuable data from part of the genome (Shi *et al*. 2005). The use of cytochrome oxidase subunit I (*COI*) has been effective in insect identification (Hebert *et al*. 2003) and has become a model in measuring evolutionary rate to examine the within-gene heterogeneity (Lunt *et al*. 1996). Furthermore, the high degree of polymorphism on *COI* (Abd-El-Samie & El Fiky 2011) also has the potential to precisely identify the invasive species and those related to biosecurity (Armstrong & Ball 2005).

The efficacy of the *COI* has been reported in many studies of *B. dorsalis*, e.g., by Yaakop *et al*. (2015a), Shi *et al*. (2010; 2012), and Schutze *et al*. (2015), among others. The effects of gamma radiation on the physiology, morphology and genetics of the pest species after the treatment need to be studied to get a holistic understanding of its efficacy in PI treatment. The optimisation process is necessary to obtain the optimum dose for the treatment which would not affect the quality of the fruits and other commodities, and consequently, for it to become the ideal technology for developing the generic treatments (IAEA 2002a; 2002b). Therefore, the aim of this study was to determine the irradiation effects on the genetics of *B. dorsalis* through nucleotide variations of the irradiated pests.

## MATERIALS AND METHODS

### Stock Culture

The stock culture of *B. dorsalis* fruit flies was reared and maintained at the Quarantine Laboratory of MARDI, Serdang, Selangor. The culture was reared at 25 ± 2°C of room temperature at 70 ± 5% relative humidity (RH), with a normal photoperiod of 12 h of daylight and 12 h of darkness, following the procedures established for *B. dorsalis* by Suhana *et al*. (2018). Eggs were collected from mature females using artificial egg oviposition plastic receptacles, which had been exposed for oviposition in a holding cage for 1 h–2 h. Eggs were then collected from the device and washed under a stream of water, and then transferred onto the rearing medium. Larvae were cultured on a wheat bran medium (artificial diet) and rearing medium following the methods of Vargas *et al*. (1984), with certain modifications. The adults were kept in a rearing cage (45 cm × 30 cm × 30 cm) made of muslin cloth and stainless-steel frame and supplied with water and food (sugar and autolysate yeast). Rotten carambola samples infested with fruit flies were collected from fruit orchards in MARDI Serdang stations representing wild fly group samples. Samples were brought to the laboratory for adult emergence and preserved in 90% ethanol prior to molecular work.

### Radiation Treatment and Grouping of Samples

The sample of third instar larvae and young puparia were prepared for the radiation treatment using Gamma Cell Biobeam GM8000 (GmBH, Germany) irradiator with Cesium-137 source at the Malaysian Nuclear Agency, Selangor, Malaysia. Before treatment, the larvae were inoculated in 237 mL enclosed plastic container (11.5 cm × 10 cm × 5.3 cm) with 70 g artificial media, and the young puparia were placed in 30 mL plastic cups with dimensions of 38 mm × 30 mm × 30 mm. They were irradiated with eight doses of gamma ray (50, 100, 150, 200, 250, 300, 350 and 400 Gy). Each treatment (dose) involved a minimum of 100 individuals per dose and a minimum of three (3) replicates. A similar group of larvae and pupae was held as the control and they were maintained in the same handling procedures, but no radiation was applied.

At the seven-day post-irradiation, the malformation of immature and adult stages of survived *B. dorsalis* were investigated and recorded. Specimens were classified into several groups based on morphological features: (1) unirradiated/control (adults, normal); (2) irradiated and with normal features (adults, normal); (3)irradiated and with abnormal features (adults, wrinkled wings); and (4) irradiated(larvae, normal). All the samples and irradiated specimens according to the doses are listed under Table 1.

### DNA Extraction and PCR Amplification

All DNA extracts from each group of individuals were obtained using the DNA NucleoSpin^®^ Insect kit (Machenery Nagel, Germany). At first, the samples were cleaned and drained thrice under 70% alcohol and then cut into small pieces using a sterile scalpel blade. The cut samples were soaked into 100 uL BE buffer to prepare the samples. These were then added with the proteinase K and 40 uL MG buffer for the lysis process. The extraction process underwent the binding, washing, drying and elution stages before the samples were ready for PCR amplification. All these steps have been followed based on the manufacturer’s protocols.

PCR amplification was conducted using the *COI* markers and primers following Folmer *et al*. (1994); *COI* F(LCO1490) (5’-GGTCAACAAATCATAAAGATATTG G-3′) and *COI* R (HCO2198) (5’-TAAACTTCAGGGTGACCAAAAAATCA-3′). A total of 25 μL reactions comprising ddH_2_0, 2.5μL PCR buffer 10× (Vivantis), 1.30 μL 50 mM MgCl_2_, 0.5 μL 10 mM dNTPs, 1.0 μL forward and reverse (5 ng/μL–10 ng/μL) were conducted using the MyGene MG96G Thermal cycler. The PCR condition comprised 40 cycles under 30 s at 95°C, 30 s at 47°C, 1 min at 72°C and final extension at 72°C for 10 min, referring to Yaakop *et al*. (2015a; 2015b) and Shariff *et al*. (2014). Products from the PCR were then electrophoresed on 1.5% agarose gel.

### DNA Sequencing, Editing and BLAST and BOLD Analyses

PCR products from the individual samples were sent to Apical Sdn. Bhd., Selangor, Malaysia for sequencing analysis. The sequences were then edited using Sequencher 4.8 software and aligned using MacClade 4.08 software and translated into protein coding gene using mtDNA code (*Drosophila melanogaster*) for detection of pseudo-heteroplasmy in determining of nuclear mitochondrial DNA (Numts) (Kowal *et al*. 2020). Basic Local Alignment Search Tool (BLAST) dan Barcode of Life Data (BOLD) analyses were conducted on the sequences to confirm the species status and no contamination (Altschul *et al*. 1990; Ratnasingham & Hebert 2007), and the results were then deposited to the Genbank.

### Distance Analysis

Distance between group samples or populations for the irradiated *B. dorsalis* was based on parameter algorithm Kimura 2-Parameter (K2P) using MEGA 7.0 software.

### Phylogenetic Analysis

NJ and MP trees were implemented to investigate the relationships between the species groups using PAUP* 4.0. The NJ was run using Kimura’s two parameter algorithm model while to get most parsimonious tree(s) (Swofford 2002), with a heuristic search (Hillis *et al*. 1996) in random addition sequences and tree bisection reconnection option for branch swapping. Support for individual clades in the tree was estimated by performing bootstrap analyses with 1,000 replications in both analyses (Felsenstein 1985).

### Haplotype and Maximum Spanning Network (MSN) Analyses

The haplotype analysis had been conducted on all the samples of *B. dorsalis* in this study using the DNA Sequence Polymorphism (DnaSP) version 5.10.01 software (Librado & Rozas 2009). Meanwhile, the MSN analysis was done to determine the clear relationships among the irradiated *B. dorsalis* from different sample groups using the Network 5.0 software.

## RESULTS

### Identity of the Species

A total of 59 samples (all sample groups) of *B. dorsalis* had been irradiated and showed no species changes based on the results of BLAST and BOLD. In the analyses, the percentage similarity as *B. dorsalis* showed between 99%–100%, similar to *B. dorsalis*. All the sequences obtained in this study were deposited in the GenBank under accession no. HSR10–HSR11 and MN193446–MN193504 (refer Table 1).

### Sequences and Alignment Analysis of *COI*

After the alignment process in all 59 sequences, 644 bp were constant (99.1%), and only 0.92% were variables. From the total variation, only four characters were found to be parsimony informative, while only two characters (0.31%) were not parsimony informative. After the alignment, a total of six sites (30, 60, 234, 282, 483 and 589) had nucleotide changes, but not translated to another protein.

### Genetic Distance

The genetic distance within and between groups is presented in Tables 2 and 3. The genetic distance between individuals in the same group is slightly higher for the unirradiated samples compared to the irradiated, however for between groups the divergence is similar and shows no difference.

### Phylogenetic Tree

**Figure 1:**
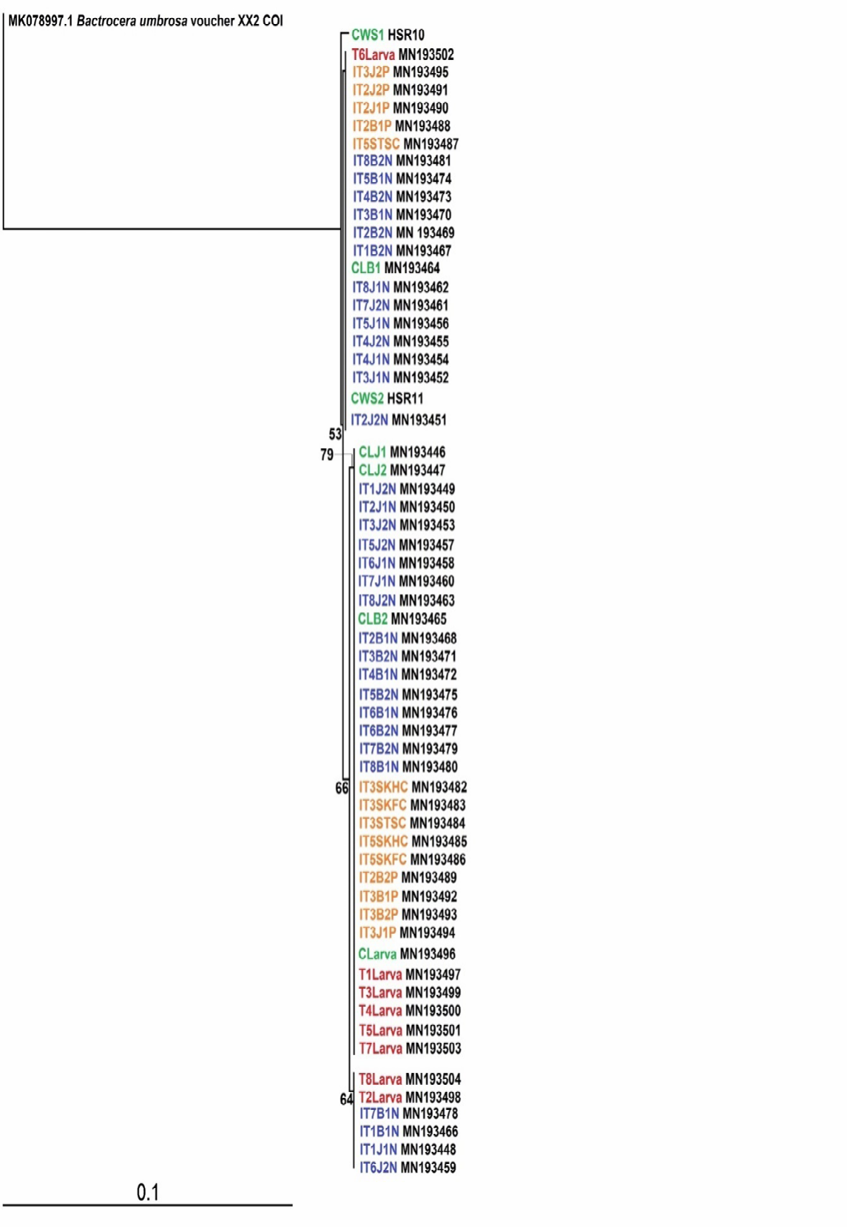
NJ tree of the *B. dorsalis* for the non-treated and treated samples. Numbers at nodes are bootstrap values (1,000 replications).

**Figure 2:**
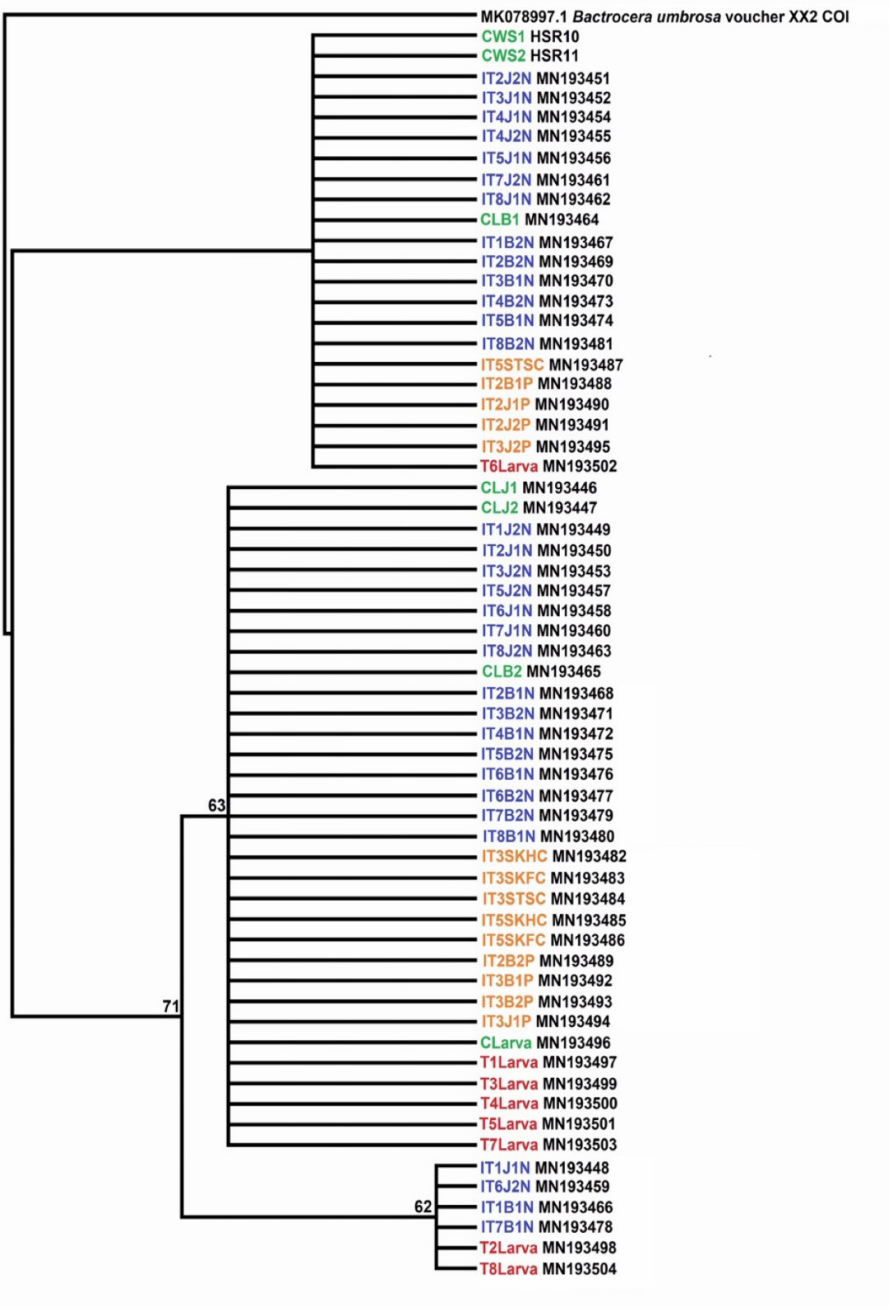
MP tree of the *B. dorsalis* for the non-treated and treated samples. Numbers at nodes are bootstrap values (1,000 replications).

The phylogenetic tree has formed soft polytomy in both trees (NJ and MP) with a mixture of individuals from different groups of treatments (Figs. 1 and 2).

### Haplotype Number and MSN Tree

A total four haplotype numbers (Tables 4 and 5) have been generated from the total individuals from all groups. Haplotype 3 (Hap3) shows the most dominant one representing the largest number of individuals, i.e., 54% (33 individuals), followed by Hap2 with 21 individuals (33%), Hap4 with 6 samples (10%), and Hap1 with only 1 sample (2%). Putative relationships (among groups) are not resolved due to hard polytomy in all the groups (treatments), which are not cladded into any specific group.

**Figure 3:**
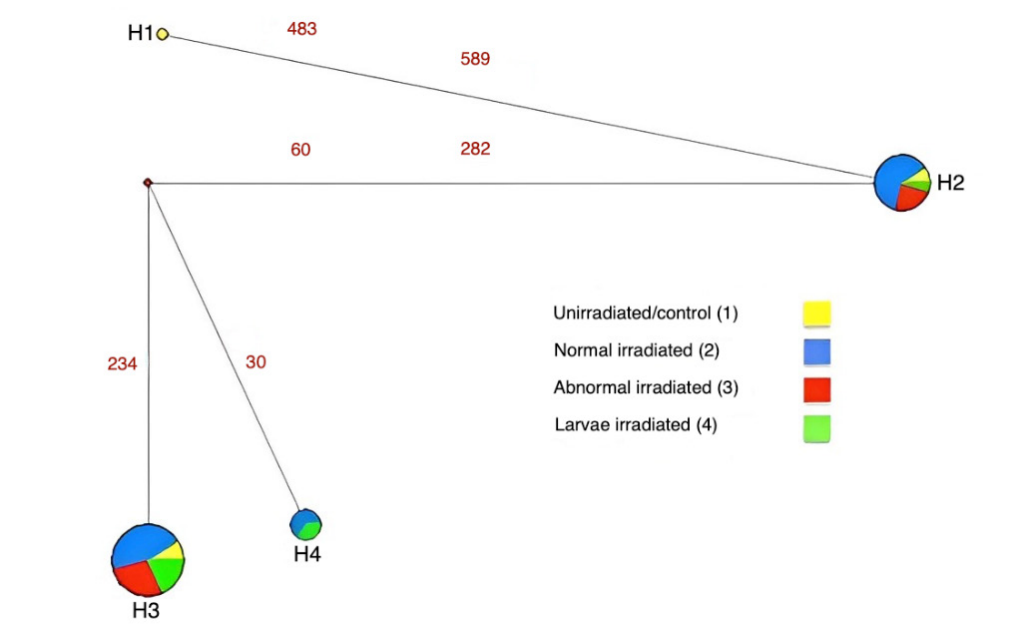
Haplotype network based on the *COI* data of *B. dorsalis* from four treatment groups.

MSN shows the clear clustering of groups based on the distribution of haplotypes and its frequency. Hap1 is the haplotype that represents only the population that has not been irradiated (wild), and Hap4 shows only the irradiated groups, while Hap2 and Hap3 represent both irradiated and unirradiated groups (Fig. 3).

## DISCUSSION

This study had been conducted to investigate the nucleotide changes in the irradiated *B. dorsalis* samples, either they were morphologically changed or not. The 50–400 Gy was the selected range because this was the dosage suggested by the USDA-APHIS (2016). Basically, the anatomy of the *B. dorsalis* in larval and adult stages had shown distinct changes with regard to the size of the reproductive organs (testis and ovary) in males and females after 10-days and 15-days irradiation with 50–100 Gy (Yusof *et al*. 2019). However, the effect on external morphology or features has never been investigated before, as well as the genetic changes in the treated samples of *B. dorsalis*.

In this study, a total of six nucleotide sites changes in both adult and larvae samples by comparing the unirradiated control and irradiated samples. The nucleotide changes however did not change the translated amino acid. The list of nucleotide changes was presented in haplotype data (Table 4), where four haplotypes were generated from this study. The changes are all transition point mutation, where it occurs between purines (G → A) and pyrimidines (C ↔ T). Low nucleotide changes were shown through the low genetic distances between unirradiated (0.004) and irradiated samples (0.002–0.003) (Tables 2 and 3). The haplotype diversity was moderately high (Hd = 0.5885), and it may refer to a stable population in a long historical evolution (Rosetti & Remis 2012).

Fourteen of the irradiated adult *B. dorsalis* showed abnormal morphologies, i.e., wrinkled wings and whitish thorax colouration in our study. According to Draz*et al*. (2016), the *B.zonata* that was irradiated between 30–90 Gy exhibited slight changes in the forewing morphology (length and width), that had resulted insignificantly reduced mating and flight ability in the field. Additionally, Draz *et al*.2016 also revealed that no significant difference in the wing’s length was evident between the irradiated and unirradiated males, but the wing’s width was slightly affected.

Four haplotypes generated in this current study, where unirradiated/control (wild) sample generates Hap1 and Hap2. Unirradiated/control (culture) samples generate Hap2 and Hap3, while irradiated samples generates Hap2, Hap3 and Hap4. Interestingly, abnormal irradiated samples generate Hap2 and Hap3, while normal and larvae irradiated samples generate Hap2, Hap3 and Hap4. The sharing haplotypes were interpreted in network figure for a clearer visual (Fig. 3). Looking into this result, we can conclude that no nucleotide changes for abnormal irradiated adult samples, because they shared the haplotypes with control (wild/culture) samples, even though the morphology of the samples changed (wrinkled wings). On the other hand, one nucleotide change occurred at 30 bp (C → T) for normal irradiated adult and irradiated larvae samples. The point mutation occurred in this study is line with previous study, where it is possible for a small-scale alteration in DNA patterns (point mutation) for the gamma rays induced mutants (Rizk *et al*. 2017).

The nucleotide changes are assumed to corroborate with the morphological and physiological changes (Sharma *et al*. 2018), whereby these changes would indicate significant mutation that would damage the genetic materials in the reproductive cells. These changes caused by the ionisation radiation, including gamma radiation, might be transferred to another generation of the target pest (Arthur *et al*. 2015). The gamma radiation has significant effect to induce dominant lethal mutations in insects for eradication purposes, which consequently would produce sterile females in the wild under the SIT programme (Robinson 2002). The latest study by Ward *et al*. (2021) has detected such a mutation in the *wp* gene that is parallel with the white pupae character in several species that are important in horticulture. Abnormal morphology of adults was present, although it does not visualise in DNA sequence in this study.

*COI* were chosen in this study due to its properties as being a good marker in barcoding analysis. The *COI* is also very effective in differentiating the cryptic species and sister species under the *B. dorsalis* complex, especially for the *B. carambolae* (Drew & Hancock 1994; Drew & Romig 2013). Furthermore, the *COI* has proven to be effective in separating several species of *Bactrocera* in Malaysia, including *B. papayae* (synonym to *B. dorsalis*), *B. carambolae, B. tau, B. latifrons*, *B. cucurbitae* and *B. umbrosa* (Chua *et al*. 2009; Yaakop *et al*. 2015a). Due to all the listed basis, amplification of *COI* is applicable for description of genetic patterns, in this point, between unirradiated and irradiated samples. The results have shown quite high haplotype diversity, and this is in line with the findings for species identification and barcoding by others (Aketarawong *et al*. 2007; Chua 2010; Muraji & Nakahara 2002; Schutze *et al*. 2015; Wan *et al*. 2011), also in invasive species for biosecurity (Armstrong & Ball 2005).

Based on the phylogenetic analyses, soft polytomy in the trees had been presented, but was unable to explain the groups’ relationships and grouping matrix. The tree topology with no single clade performed served to verify that no clustering of any group had been constructed. It was necessary then to determine the distance between groups, haplotype distribution and haplotype diversity of among the *B. dorsalis* groups. The hypothesis to observe for any variations in genetic features between the gamma irradiated and unirradiated populations can be conducted through this process. Studies by Syarifah-Zulaikha *et al*. (2021) and Badrulisham *et al*. (2021) had shown that a mixture of pest individuals from all regions in Peninsular Malaysia exhibited rather low separation of populations, despite a combination of data on genes being implemented for the analysis.

The variation of nucleotides after irradiation is one of the parameters to measure the protein changes, whereas the reaction due to radiation is the main factor in resistance and effective irradiation (Suman *et al*. 2015). The nucleotide changes are significant for determining the protein changes that directly change the peptide leading to the development of a new species and towards speciation. In this context, the change into a new species in the horticulture and postharvest stages would probably create more adverse impacts caused by the newly developed mutant species (Carlin 2011). Conversely, the development of low survival and reproduction capacity in the evolution of *B. dorsalis* would be good for the pest control purposes of this species.

These findings are very significant for the phytosanitary process during the postharvest, as well as for the quarantine system towards minimising the spread of the *B. dorsalis* pest worldwide from the trading activities. Although the data obtained are quite fundamental, however, its implication is very significant in understanding the effects of gamma irradiation on *B. dorsalis*, especially on the larval or pupal stages that inhabit the inside parts of the fruit. Furthermore, understanding the effect on the nucleotide changes would be very useful when applied on the eggs, which is an extremely difficult stage to control. Generally, mutation that has occurred on the DNA fragment of the irradiated *B. dorsalis* needs further and more detailed study regarding its survival and reproduction in its evolution as a horticultural pest worldwide. In addition, the gamma irradiation doses can be further explored as the pest’s fitness also play significant roles towards effective phytosanitary efforts.

## Figures and Tables

**Table 1:** List of samples of *B. dorsalis* used in this study (code of samples, location, features, GenBank accession no. and treatment group (1) not irradiated/control; (2) normal irradiated; (3)abnormal irradiated and (4) larvae irradiated) based on *COI* data.

No.	Code samples	Location	Treatment (Gamma dose)	Feature	Gender	Treatment groups	GenBank accession no.	%	

								BLAST	BOLD
1	CWS1	Selangor: Serdang	-	Normal	-	1	HSR10	99	99
2	CWS2	Selangor: Serdang	-	Normal	-	1	HSR11	99	100
3	CLJ1	Selangor: Serdang	-	Normal	Male	1	MN193446	100	100
4	CLJ2	Selangor: Serdang	-	Normal	Male	1	MN193447	100	100
5	CLB1	Selangor: Serdang	-	Normal	Female	1	MN193464	100	100
6	CLB2	Selangor: Serdang	-	Normal	Female	1	MN193465	100	100
7	IT1J1N	Selangor: Serdang	50	Normal	Male	2	MN193448	100	100
8	IT1J2N	Selangor: Serdang	50	Normal	Male	2	MN193449	100	100
9	IT2J1N	Selangor: Serdang	100	Normal	Male	2	MN193450	100	100
10	IT2J2N	Selangor: Serdang	100	Normal	Male	2	MN193451	100	100
11	IT3J1N	Selangor: Serdang	150	Normal	Male	2	MN193452	100	100
12	IT3J2N	Selangor: Serdang	150	Normal	Male	2	MN193453	100	100
13	IT4J1N	Selangor: Serdang	200	Normal	Male	2	MN193454	100	100
14	IT4J2N	Selangor: Serdang	200	Normal	Male	2	MN193455	100	100
15	IT5J1N	Selangor: Serdang	250	Normal	Male	2	MN193456	100	100
16	IT5J2N	Selangor: Serdang	250	Normal	Male	2	MN193457	100	100
17	IT6J1N	Selangor: Serdang	300	Normal	Male	2	MN193458	100	100
	IT6J2N	Selangor: Serdang	300	Normal	Male	2	MN193459	100	100
18	IT7J2N	Selangor: Serdang	350	Normal	Male	2	MN193461	100	100
	IT7J1N	Selangor: Serdang	350	Normal	Male	2	MN193460	100	100
19	IT8J1N	Selangor: Serdang	400	Normal	Male	2	MN193462	100	100
20	IT8J2N	Selangor: Serdang	400	Normal	Male	2	MN193463	100	100
21	IT1B1N	Selangor: Serdang	50	Normal	Female	2	MN193466	100	100
22	IT1B2N	Selangor: Serdang	50	Normal	Female	2	MN193467	100	100
23	IT2B1N	Selangor: Serdang	100	Normal	Female	2	MN193468	100	100
24	IT2B2N	Selangor: Serdang	100	Normal	Female	2	MN193469	100	100
25	IT3B1N	Selangor: Serdang	150	Normal	Female	2	MN193470	100	100
26	IT3B2N	Selangor: Serdang	150	Normal	Female	2	MN193471	100	100
27	IT4B1N	Selangor: Serdang	200	Normal	Female	2	MN193472	100	100
28	IT4B2N	Selangor: Serdang	200	Normal	Female	2	MN193473	100	100
29	IT5B1N	Selangor: Serdang	250	Normal	Female	2	MN193474	100	100
30	IT5B2N	Selangor: Serdang	250	Normal	Female	2	MN193475	100	100
31	IT6B1N	Selangor: Serdang	300	Normal	Female	2	MN193476	100	100
32	IT6B2N	Selangor: Serdang	300	Normal	Female	2	MN193477	100	100
33	IT7B1N	Selangor: Serdang	350	Normal	Female	2	MN193478	100	100
34	IT7B2N	Selangor: Serdang	350	Normal	Female	2	MN193479	100	100
35	IT8B1N	Selangor: Serdang	400	Normal	Female	2	MN193480	100	100
36	IT8B2N	Selangor: Serdang	400	Normal	Female	2	MN193481	100	100
37	IT3SKHC	Selangor: Serdang	150	Wrinkle wing (half)	Male	3	MN193482	100	100
38	IT3SKFC	Selangor: Serdang	150	Wrinkle wings (full)	Male	3	MN193483	100	100
39	IT3STSC	Selangor: Serdang	150	Wings asymmetry	Male	3	MN193484	100	100
40	IT5SKHC	Selangor: Serdang	250	Wrinkle wings (half)	Male	3	MN193485	100	100
41	IT5SKFC	Selangor: Serdang	250	Wrinkle wing (full)	Male	3	MN193486	100	100
42	IT5STSC	Selangor: Serdang	250	Wings asymmetry	Male	3	MN193487	100	100
43	IT2B1P	Selangor: Serdang	100	Whitish thorax	Female	3	MN193488	100	100
44	IT2B2P	Selangor: Serdang	100	Whitish thorax	Female	3	MN193489	100	100
45	IT2J1P	Selangor: Serdang	100	Whitish thorax	Male	3	MN193490	100	100
46	IT2J2P	Selangor: Serdang	100	Whitish thorax	Male	3	MN193491	100	100
47	IT3B1P	Selangor: Serdang	150	Whitish thorax	Female	3	MN193492	100	100
48	IT3B2P	Selangor: Serdang	150	Whitish thorax	Female	3	MN193493	100	100
49	IT3J1P	Selangor: Serdang	150	Whitish thorax	Male	3	MN193494	100	100
50	IT3J2P	Selangor: Serdang	150	Whitish thorax	Male	3	MN193495	100	100
51	Clarva	Selangor: Serdang	0	Normal	-	4	MN193496	100	100
52	T1Larva	Selangor: Serdang	50	Normal	-	4	MN193497	100	100
53	T2Larva	Selangor: Serdang	100	Normal	-	4	MN193498	100	100
54	T3Larva	Selangor: Serdang	150	Normal	-	4	MN193499	100	100
55	T4Larva	Selangor: Serdang	200	Normal	-	4	MN193500	100	100
56	T5Larva	Selangor: Serdang	250	Normal	-	4	MN193501	100	100
57	T6Larva	Selangor: Serdang	300	Normal	-	4	MN193502	100	100
58	T7Larva	Selangor: Serdang	350	Normal	-	4	MN193503	100	100
59	T8Larva	Selangor: Serdang	400	Normal	-	4	MN193504	100	100

**Table 2:** Genetic distance of *B. dorsalis* individuals among treatment groups based on *COI* data.

Sample groups	Treatment group no.	Distance
Unirradiated/ control	1	0.004
Normal irradiated	2	0.003
Abnormal irradiated	3	0.002
Larvae irradiated	4	0.002

**Table 3:** Matrix genetic distance between *Bactrocera dorsalis* among treatment groups based on *COI* data.

Samples	Non-irradiated/Control (1)	Normal irradiated (2)	Abnormal irradiated (3)	Larvae irradiated (4)
Unirradiated/control (1)	-	-	-	-
Normal irradiated (2)	0.003	-	-	-
Abnormal irradiated (3)	0.003	0.002	-	-
Larvae irradiated (4)	0.003	0.002	0.002	-

**Table 4:** Haplotype diversity based on 650 bp of *COI* sequences of the *B. dorsalis* individuals.

Group/Site			2	2	4	5	Total samples
	3	6	3	8	8	8	
	0	0	4	2	3	9	
Haplotype 1	C	T	C	C	G	T	1 (2%)
Haplotype 2	C	C	C	T	G	T	21 (34%)
Haplotype 3	C	C	T	T	A	C	33 (54%)
Haplotype 4	T	C	C	T	A	C	6 (10%)

**Table 5:** Haplotype distribution on the *B. dorsalis* individuals based on *COI* data.

Samples group	Haplotype			

	1	2	3	4
Unirradiated/control (1) (wild)	1	1	-	-
Unirradiated/control (1) (culture)	-	1	4	-
Normal irradiated (2)	-	13	15	4
Abnormal irradiated (3)	-	5	9	-
Larvae irradiated (4)	-	1	5	2
